# The Tumor Stroma of Squamous Cell Carcinoma: A Complex Environment That Fuels Cancer Progression

**DOI:** 10.3390/cancers16091727

**Published:** 2024-04-29

**Authors:** Alexandra Buruiană, Bogdan-Alexandru Gheban, Ioana-Andreea Gheban-Roșca, Carmen Georgiu, Doința Crișan, Maria Crișan

**Affiliations:** 1 Department of Pathology, Iuliu Haţieganu University of Medicine and Pharmacy, 400012 Cluj-Napoca, Romania; buruiana.alexandra@umfcluj.ro (A.B.); cgeorgiu@umfcluj.ro (C.G.); dcrisan@umfcluj.ro (D.C.); 2 Department of Histology, Iuliu Haţieganu University of Medicine and Pharmacy, 400012 Cluj-Napoca, Romania; maria.crisan@umfcluj.ro; 3 Emergency Clinical County Hospital, 400347 Cluj-Napoca, Romania; 4 Department of Medical Informatics and Biostatistics, Iuliu Hațieganu University of Medicine and Pharmacy, 400129 Cluj-Napoca, Romania; andreea.gheban-rosca@umfcluj.ro

**Keywords:** cutaneous squamous cell carcinoma, tumor microenvironment, stroma, extracellular matrix, immune cells, therapy

## Abstract

**Simple Summary:**

Despite the high prevalence of cutaneous squamous cell carcinoma (cSCC), one of the most frequent human cancers, the tumor microenvironment (TME) of this specific cancer remains understudied. This review aims to comprehensively characterize the cSCC tumor stroma, examining its cellular and molecular components. We will delve into the TME’s dynamic role in tumor progression, going beyond its traditionally viewed role as a passive element. Furthermore, the review will explore the TME’s significant impact on cSCC therapy, highlighting its potential to both hinder treatment efficacy and offer targets for groundbreaking therapeutic approaches.

**Abstract:**

The tumor microenvironment (TME), a complex assembly of cellular and extracellular matrix (ECM) components, plays a crucial role in driving tumor progression, shaping treatment responses, and influencing metastasis. This narrative review focuses on the cutaneous squamous cell carcinoma (cSCC) tumor stroma, highlighting its key constituents and their dynamic contributions. We examine how significant changes within the cSCC ECM—specifically, alterations in fibronectin, hyaluronic acid, laminins, proteoglycans, and collagens—promote cancer progression, metastasis, and drug resistance. The cellular composition of the cSCC TME is also explored, detailing the intricate interplay of cancer-associated fibroblasts (CAFs), mesenchymal stem cells (MSCs), endothelial cells, pericytes, adipocytes, and various immune cell populations. These diverse players modulate tumor development, angiogenesis, and immune responses. Finally, we emphasize the TME’s potential as a therapeutic target. Emerging strategies discussed in this review include harnessing the immune system (adoptive cell transfer, checkpoint blockade), hindering tumor angiogenesis, disrupting CAF activity, and manipulating ECM components. These approaches underscore the vital role that deciphering TME interactions plays in advancing cSCC therapy. Further research illuminating these complex relationships will uncover new avenues for developing more effective treatments for cSCC.

## 1. Introduction

**Figure 1:**
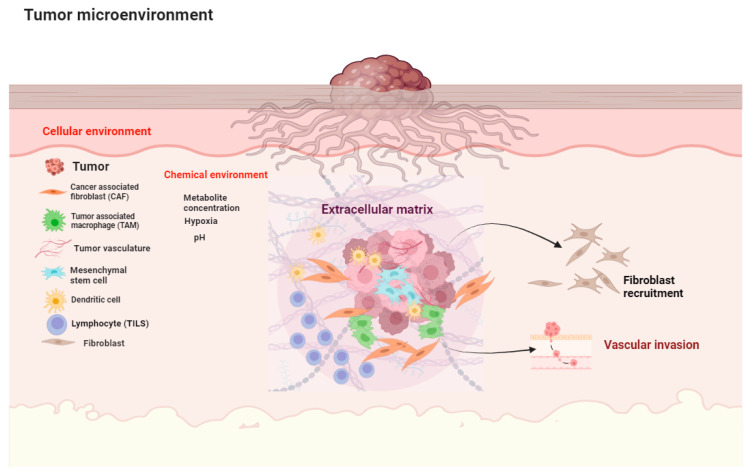
The squamous cell carcinoma tumor microenvironment (TME). Detailed schematic representation highlighting key components and processes. Cellular components include cancer-associated fibroblasts (CAFs), tumor-associated macrophages (TAMs), mesenchymal stem cells (MSCs), dendritic cells, and tumor-infiltrating lymphocytes (TILs). The extracellular matrix (ECM) is depicted, including structural proteins such as collagen, fibronectin and laminins. Key processes such as fibroblast recruitment, vascular invasion, and the establishment of chemical gradients (hypoxia, pH, metabolites) are highlighted.

The tumor stroma, or tumor microenvironment (TME), is the collection of non-cancerous cells, blood vessels, and extracellular matrix (ECM) that surrounds and interacts with cancer cells [1]. It plays a complex and significant role in tumor progression, invasion, and metastasis [2] (Figure 1).

Understanding the composition and function of the tumor stroma is an important area of cancer research [3]. This knowledge is being used to develop new therapies that target the tumor stroma with the aim of improving the effectiveness of cancer treatment.

The two main components of the tumoral stroma are the extracellular matrix (ECM) and a cellular component. The extracellular matrix is a complex network of proteins and sugars that provides structural support to the tumor and plays a role in cell signaling and communication, thus playing an important role in cancer development and progression [4]. The main components of the ECM in tumor stroma include collagens, laminins, fibronectins, and proteoglycans.

The cellular component includes cancer-associated fibroblasts (CAFs), mesenchymal stem cells (MSCs), endothelial cells (which line blood vessels), pericytes (which wrap around blood vessels), immune cells, and adipocytes (fat cells) [5]. CAFs are the most abundant cell type in the tumor stroma and are activated fibroblasts that promote tumor growth, invasion, and metastasis [6]. They produce various factors that support tumor cells, such as growth factors, cytokines, and extracellular matrix (ECM) proteins [7]. The MSCs are stem cells that can differentiate into various types of cells, including CAFs [8]. These cells can suppress the immune system and release growth factors that help to support tumor growth and tumor spread [9]. Endothelial cells line the blood vessels within the tumor and are responsible for delivering nutrients and oxygen to tumor cells [10]. By expressing vascular endothelial growth factor (VEGF), the blood vessels maintain increased permeability [11], resulting in leakage that contributes to tumor growth and spread [12]. Pericytes, which are wrapped around blood vessels, help to regulate blood flow [13]. They can also promote tumor growth and metastasis [14] by promoting angiogenesis [15], vascular remodeling [16], protecting the tumor vessels from damage caused by anti-angiogenic therapies, and by promoting tumor survival and resistance to treatment [17]. They can also interact with tumor cells through cell-to-cell contact and thus support tumor cell survival and proliferation [18] or even hinder the body’s ability to fight the cancer [18] by creating an immunosuppressive environment within the tumor microenvironment [19]. Lastly, but not least importantly, they can facilitate tumor cell invasion, migration, and metastasis by detaching from the blood vessels and infiltrating the tumor stroma, where they interact with the cancer cells directly and induce the release of enzymes that will eventually break down the ECM [20], creating channels within the tumoral stroma that can serve as veritable “highways” for the tumor cells to navigate [21]. The tumor stroma can contain a variety of immune cells, including both tumor-promoting and tumor-suppressing cells [2]. The balance between these two types of cells can determine the effectiveness of the immune system in attacking the tumor. Fat cells can store energy and produce various factors that promote tumor growth and metastasis [22].

The heterogeneous composition of the tumor stroma is influenced by both cancer type and stage, potentially modulating treatment response. A common characteristic is a dense, desmoplastic stroma [23]. Desmoplasia denotes the excessive deposition of fibrous connective tissue, particularly collagen, within the tumor’s microenvironment [24], which is a reaction often induced by the tumor cells themselves [25]. This dense stromal composition can paradoxically affect tumor progression. Although it may serve to physically restrain tumor expansion, desmoplasia can also impede drug delivery and limit immune cell access to the tumor site [24,26]. Morphologically, desmoplasia may be categorized as immature (featuring loosely arranged collagen fibers, inflammatory cells, and vasculature), mature (exhibiting densely organized mature collagen fibers), or intermediate (a hybrid state) [27].

## 2. Aim

This review aims to comprehensively characterize the tumor stroma of cutaneous squamous cell carcinoma (cSCC) and its constituent cellular and molecular components. Beyond its traditional perception as a passive bystander, the tumor stroma has emerged as a complex and dynamic microenvironment that actively supports tumor cell growth, migration, invasiveness, and metastasis. Furthermore, we emphasize the critical role of the tumor microenvironment (TME) in cSCC therapy. We will highlight the TME’s capacity to both shield tumor cells from therapeutic interventions and offer potential targets for novel therapeutic strategies.

## 3. Extracellular Matrix Components in Cutaneous Squamous Cell Carcinoma

### 3.1. Fibronectin

Fibronectin is a large glycoprotein that acts as a bridge between cells and other ECM components [28]. It helps with cell adhesion, migration, and proliferation [29]. Fibronectin is not a single uniform chain but rather a collection of modules or domains, each with specific functions [30]. Due to its modular design and flexibility, fibronectin does not have a well-defined, rigid 3D structure. It is more like a dynamic scaffold, able to adapt its conformation to interact with various partners within the ECM [31].

Fibronectin in cSCC is a potential player with multifaceted roles. It might influence tumor progression and invasion by aiding in cancer cell adhesion and migration within the cSCC stroma [32]. Additionally, studies suggest that fibronectin interacts with other ECM components in cSCC, potentially remodeling the tumor microenvironment and impacting tumor growth, behavior, and treatment response [33]. Interestingly, some research suggests a link between chronic wounds (where fibronectin is involved in healing) and cSCC development, although the exact mechanisms require further investigation [34,35].

More studies are needed to definitively understand how fibronectin contributes to cSCC development and progression.

### 3.2. Hyaluronic Acid

Hyaluronic acid is a glycosaminoglycan that contributes to hydration, cell signaling, and matrix organization [36]. In normal skin, its functions are to retain water, contribute to hydration, give suppleness and elasticity, and aid in wound healing [37,38].

The role of hyaluronic acid (HA) in cancer is complex, and it can actually have opposing effects depending on the context [39]. Our understanding of HA’s role in SCC is evolving. Research suggests that it exhibits both anti-tumorigenic and pro-tumorigenic effects in this type of cancer. Studies have implicated HA in promoting tumorigenesis through its interactions with cell surface receptors. These interactions trigger downstream signaling cascades that culminate in enhanced cancer cell proliferation, migration, and invasion [40], thus contributing to tumor growth and the spread of cancer cells.

Hyaluronic acid is not just a structural component in tumors; it actively promotes their growth by influencing blood vessel formation (angiogenesis) [41]. It interacts with endothelial cells (blood vessel-lining cells), triggering pathways that make them multiply, migrate, and form new vessels [42]. Additionally, HA regulates critical growth factors like VEGF, which further promotes blood vessel formation [43]. HA plays a crucial role in ECM remodeling, making it easier for endothelial cells to move and build new blood vessels and so fueling tumor expansion. Finally, hyaluronic acid’s influence on ECM remodeling creates a permissive environment for endothelial cell migration and blood vessel formation, thus promoting tumor growth [44].

The relationship between hyaluronic acid (HA) and drug resistance is a double-edged sword [45]. It can promote drug resistance by two mechanisms. Firstly, HA can create a physical barrier when present at high levels in the tumor microenvironment, forming a dense, gel-like network. This barrier hinders the penetration and delivery of chemotherapy drugs to cancer cells [46]. Also, by interacting with receptors like CD44 on cancer cells, it can activate pathways that promote cell survival and resistance to drugs [47]. On the other hand, HA can be used as a carrier for chemotherapy drugs [48]. By targeting receptors on cancer cells, HA-based carriers can deliver drugs more effectively and overcome resistance [49].

Studies have suggested that HA can also have an anti-tumorigenic effect. It is suggested that HMW-HA, a specific form of hyaluronic acid with a larger molecular size, might actually act against cancer progression. It may strengthen cell-to-cell connections and decrease the permeability of the extracellular matrix (ECM), making it harder for cancer cells to invade surrounding tissues [40]. Additionally, HA may trigger apoptosis, a form of programmed cell death, in cancer cells [50].

### 3.3. Laminins

Laminins are crucial heterotrimeric glycoproteins found in the extracellular matrix of all animals [51]. They play essential roles in various biological processes, particularly cell adhesion, differentiation, migration, and signaling [52]. Laminin molecules are composed of three different chains—alpha, beta, and gamma—forming various combinations that create numerous laminin isoforms with potentially unique functions [53]. Being a glycoprotein means that laminins can attach to glycans and influence its interactions with other molecules [54]. 

Laminins are a significant component of the basement membrane, which is a specialized layer within the extracellular matrix that provides structural support and separates cells from surrounding tissues [55]. In simpler terms, laminins are versatile proteins [54] with three chain subunits that function like molecular glue in the basement membrane, influencing how cells interact with their environment [55,56].

**Figure 2:**
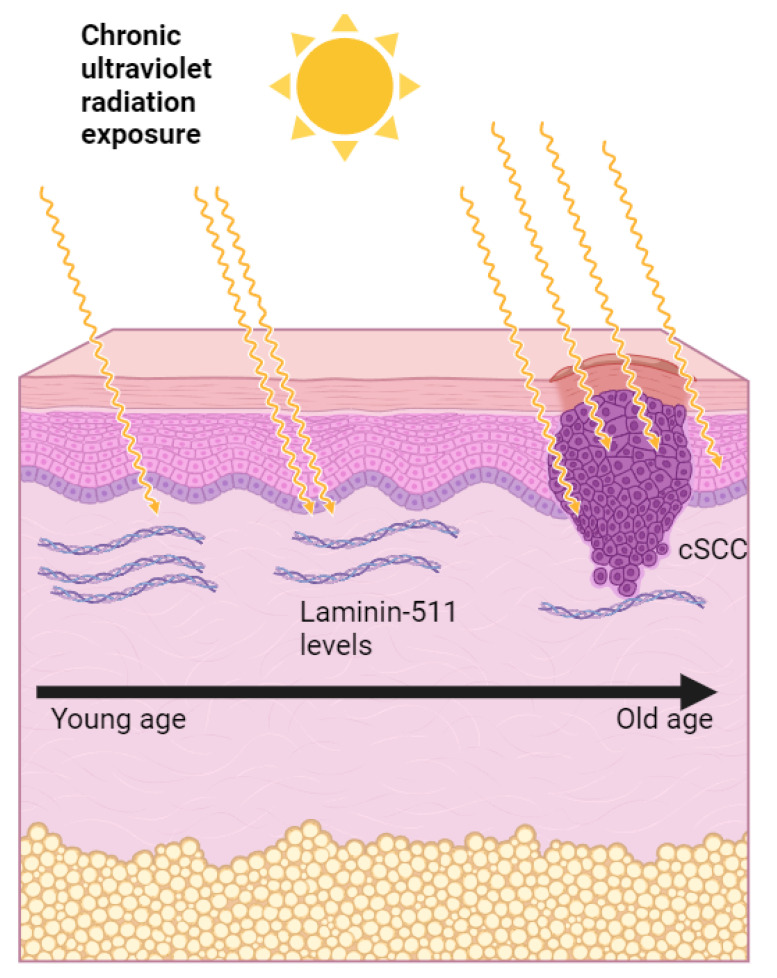
Potential link between reduced laminin-511 levels and cutaneous squamous cell carcinoma development (cSCC) due to UV damage. This diagram illustrates the effects of cumulative ultraviolet (UV) exposure (represented in the image with yellow arrows) on laminin-511 levels within the dermal–epidermal junction (DEJ). The illustration compares skin with minimal UV exposure to skin with chronic sun exposure. Recent studies suggest that accumulated UV damage, the hallmark feature of photoaging, leads to a reduction in laminin-511, a protein critical for cell adhesion within the DEJ [63]. This decrease may be relevant to the development of cutaneous squamous cell carcinoma (cSCC), a skin cancer that is more prevalent in sun-exposed areas of elderly individuals.

In healthy human skin, laminin-332 and laminin-511 are considered major laminin isoforms [57]. They play crucial roles in maintaining the structure and function of the skin [58], both playing essential roles in cell adhesion by helping to anchor the keratinocytes to the underlying basement membrane, thus maintaining structural integrity [57], and in cellular communication by interacting with cell receptors, thus influencing cell growth, differentiation, and migration [59]. They are both primarily found in the dermal–epidermal junction of the skin [60]. Studies have shown that laminin-332 may be upregulated in wound healing, potentially aiding the migration of keratinocytes during re-epithelialization [61], whereas the laminin-511 isoform might play a role in hair follicle growth based on its upregulation during the anagen phase [60]. Research also suggests that high levels of laminin-332 might correlate with increased tumor invasiveness in cutaneous squamous cell carcinoma (cSCC) [62]. Recent research indicates that photoaging, induced by chronic UV irradiation, leads to a reduction in laminin-511 levels within the dermal–epidermal junction [63]. Given that cutaneous squamous cell carcinoma (cSCC) predominantly arises in elderly individuals and on sun-exposed skin, it is plausible to hypothesize that reduced levels of the laminin-511 isoform may be observed in cSCC lesions. Nonetheless, further investigations are necessary to reach a definitive conclusion regarding this potential association (Figure 2).

Laminin-332 and laminin-511 are essential contributors to healthy skin structure and function. However, their specific roles and potential variations in expression during development, wound healing, or diseases like cSCC require further exploration.

### 3.4. Proteoglycans

Proteoglycans are complex molecules consisting of a core protein with one or more covalently attached glycosaminoglycan (GAG) chains [64]. They are essential components of the extracellular matrix, providing structural support and hydration and playing a role in cell signaling. 

In the tumor stroma, they exert complex and multi-layered influences [65]. Firstly, proteoglycans contribute to the formation of a dense and altered extracellular matrix (ECM), which provides a supportive structural framework that facilitates cancer cell proliferation and dissemination [66]. Additionally, this altered ECM can impede the effective infiltration and function of immune cells within the tumor microenvironment, thus hindering them from effectively attacking the tumor [3]. Secondly, they can behave like signaling hubs through being able to bind to and interact with various growth factors, cytokines, and other signaling molecules within the tumor microenvironment [67]. This can influence cancer cell survival, growth, angiogenesis, and metastasis. Lastly, proteoglycans can alter the mechanics of the ECM, as their abundance in the tumor stroma can increase its stiffness [67]. This stiffer environment can promote cancer cell aggression and may make the tumor more resistant to some treatments [68,69].

Decorin, versican, and biglycan are the major proteoglycans found within the human dermis [70]. Decorin, the most abundant of these proteoglycans, interacts with collagen fibers and influences their assembly and diameter, thereby contributing to skin elasticity [71,72]. Versican, another crucial component of the ECM, plays a significant role in skin hydration and resilience [73]. Biglycan is involved in collagen fibrillogenesis and may also contribute to wound-healing processes within the skin [74].

Studies show that versican can be overexpressed in the stroma of some squamous cancers of the esophagus [75] and pharynx [76]. Its abundance in the skin and its roles in tumor growth [77], inflammation [78], angiogenesis [79], and alteration of the physical properties of the tumor environment make it plausible to find it upregulated in cSCC as well. Interestingly, versican can bind to various types of immune cells, including macrophages and T cells [80]. This binding interaction can trigger signaling pathways within these immune cells, leading to their activation and the release of pro-inflammatory molecules (cytokines and chemokines) [79]. Versican is also responsible for altering the ECM by acting like a magnet for inflammatory cells and recruiting them to the tumor microenvironment [81]. These recruited cells can further amplify the inflammatory response. Versican can bind to and activate Toll-like receptors (TLRs), mainly TLR2, on innate immune cells like macrophages [82]. This activation further amplifies inflammatory signaling, contributing to chronic inflammation associated with tumor growth [83] and even metastasis [82]. 

Unlike versican, which consistently promotes tumor progression [77,78,79], decorin appears to have a more multifaceted role in cSCC. Research points to it potentially acting as a tumor suppressor [84] and also as a tumor promoter when its levels are decreased [85]. Decorin can bind to and sequester various growth factors, including TGF-beta, which can limit availability of such factors to cancer cells and hinder tumor growth [84], and some studies suggest that decorin may hinder angiogenesis, which is a crucial process for tumor survival [86] as well as new lymphatic vessel formation [87]. There is some interest in exploring whether delivering decorin or boosting its expression within the tumor stroma could have a therapeutic effect in some cSCC cases [88].

### 3.5. Collagens

Collagens are structural proteins that act as the main building blocks for various connective tissues in the body, including skin, bones, tendons, ligaments, and cartilage [89]. They provide strength, elasticity, and support to these tissues [90]. There are at least 28 distinct types of collagen that have been identified in the human body [89]. These types are classified into five groups based on their structure and the assemblies they form [91]. Type I collagen is the most abundant type and it is found in skin tendons, bones, ligaments, and other organs [92]. Often found alongside type I collagen, type III collagen plays a supporting role in skin structure, is important for elasticity, and plays a role in wound healing [93]. The natural production of collagens declines with age; thus, lower levels of collagen I and III contribute to a loss in skin elasticity and increase in wrinkle formation [94]. Collagen IV is found in the skin as well, albeit in smaller amounts than the two aforementioned types; its presence in the basement membrane, a thin layer separating the dermis (inner layer of skin) and the epidermis (outer layer), means that it plays a critical role in anchoring and supporting the skin’s structure [95], thus maintain the overall integrity and function of the skin. Collagen V is also present in the skin [96] and plays a regulatory role in the assembly of other collagen fibrils, particularly type I collagen [97].

In the TME, the fibrillar collagens (types I, III, and V) are the most abundant collagen types. They form thick, strong fibers providing structural support. Increased deposition and stiffness of fibrillar collagens can act as a physical barrier for drug delivery and immune cell infiltration [98]. Network-forming collagens such as type IV have typically higher levels, thus promoting tumor growth and invasiveness [99] by interacting with proteins called integrins on the surface of cancer cells [100], triggering signaling pathways that can promote cell survival, proliferation, and migration, enhancing the invasive behavior of cancer cells [101]; by protecting the cancer cell from immune attack by creating a physical barrier, they hinder the access of immune cells to the tumor and shield cancer cells from the immune response [102]. Type XII and XIV collagens, called “Fibril Associated Collagens with Interrupted Triple Helices” [103], interact in the TME with fibrillar collagens and other ECM components and influencing ECM organization [104]. 

The composition and organization of collagens within the tumor microenvironment significantly influence tumor progression by promoting growth, facilitating invasion and metastasis, modulating the immune response, and contributing to drug resistance. Stiff, dense networks of collagens can promote tumor cell proliferation and survival [105], and cancer cells are able to use collagen fibers as “highways” to invade surrounding tissues [106]. The collagen degradation induced by enzymes like matrix metalloproteinases (MMPs) helps cancer cells break free and spread to distant sites [107]. A dense collagen matrix poses a significant obstacle to immune cell infiltration and drug delivery to the tumor site, thereby compromising both immune-mediated tumor destruction [108] and the efficacy of therapeutic agents [109].

Some authors have noted that in the progression of keratinocyte intraepidermal neoplasia (KIN) toward invasive cutaneous squamous cell carcinoma, the levels of type I collagen steadily increase, particularly in the papillary dermis that is directly beneath the progressing tumor [110]. In the same study, an increase in the number of fibroblasts in the skin was detected as the KIN progressed to cSCC [110]. The findings of another study implicate the disruption of several interconnected biological processes due to the absence of type VII collagen, including chronic inflammation stemming from impaired wound healing, dysregulation of TGF-β signaling cascades, and abnormalities in immune function, all of which could contribute to the development of cSCC in dystrophic epidermolysis bullosa (DEB) patients [111].

## 4. Cellular Components of the TME in Cutaneous Squamous Cell Carcinoma

### 4.1. Cancer-Associated Fibroblasts (CAFs)

In contrast to the tissue-protective functions of normal fibroblasts in wound healing and structural maintenance [112], cancer-associated fibroblasts (CAFs) arise within the tumor microenvironment (TME) and directly support tumor growth, progression, and metastasis [113]. 

The primary source of CAFs is considered to be resident fibroblasts that undergo transformation [114], which is triggered by growth factors secreted by cancer cells (e.g., TGF-β, Platelet-Derived Growth Factor) [115], inflammatory cytokines such as IL-1β, IL-6, and TNF-α [7,116], hypoxia [117], and direct interaction with cancer cells [118], although the are other sources of CAFs such as the bone marrow [119] and adipose tissue [120]. Endothelial cells can potentially transform into fibroblast-like cells, thereby adding to the CAF population [121]. While it is less common, there is some evidence suggesting that, in some instances, epithelial cells might change to become CAFs [122]. Evidence suggests that the disparate cellular lineages of CAFs confer substantial heterogeneity upon this population. This heterogeneity may manifest as divergent properties and specialized functions within the context of the tumor microenvironment [123]. Understanding the developmental pathways leading to different CAF subtypes could open avenues for preventing their formation in the first place [124].

CAFs influence cSCC development and progression in several ways. One of these way is by promoting tumor growth by secreting growth factors [122], such as transforming growth factor-beta (TGF-β) [125]. CAFs help cancer cells break away from the primary tumor and migrate to other parts of the body by secreting enzymes that degrade the ECM, such as matrix metalloproteinases (MMPs) [126], and by supporting the process of angiogenesis through the secretion of VEGF among other factors. The CAF subtypes are largely absent in pre-cancerous lesions but become prominent in cSCC tumors, highlighting their roles in active tumor progression [124]. 

The presence of two functionally divergent CAF subtypes has been elucidated in cSCC: immunomodulatory CAFs, which mediate the suppression of anti-tumor immune responses within the TME, and matrix-remodeling CAFs, which promote tumor invasion and metastatic potential through restructuring the ECM [124].

CAFs can contribute to drug resistance in cancer cells, making treatments less effective [127]. Through the secretion of matrix metalloproteinases (MMPs), CAFs induce structural remodeling of the extracellular matrix (ECM) [113]. This results in increased ECM density and stiffness [128], creating a physical barrier that reduces drug penetration into the tumor [129]. The excess ECM deposition caused by CAFs, termed desmoplasia, which is a hallmark of many tumor types, including cSCC, is a significant contributor to drug resistance [130]. The effectiveness of immunotherapy is also hindered, as CAFs are able to release molecules that suppress anti-tumor immune cells like T cells [131]. CAFs can induce resistance to photodynamic therapy (PDT) in cutaneous squamous cell carcinoma via the secretion of TGFβ1, with response heterogeneity observed across different cSCC cell lines [125]. 

CAFs in recurrent cSCC actively communicate with tumor cells, potentially driving the related EMT changes and promoting proliferation and metastasis. Thus, a unique population of tumor cells with EMT features emerges in recurrent cSCC [132].

### 4.2. Mesenchymal Stem Cells (MSCs)

Mesenchymal stem cells are multipotent stem cells [133] that can differentiate into various cell types, including osteoblasts, chondrocytes, and adipocytes [134]. They are found in various tissues, including bone marrow, adipose tissue, and umbilical cord [135], and have immunomodulatory and regenerative properties, making them an attractive potential tool for various medical applications [136].

MSCs have a complex relationship with cancer, and their role can be both beneficial and detrimental depending on the circumstances. In some cases, MSCs within the tumor microenvironment can promote cancer progression [137], while in others they can exhibit anti-tumor effects [138]. They may support tumor growth by secreting growth factors such as [139] TGF-β, vascular endothelial growth factor (VEGF), fibroblast growth factor (FGF), and others. Studies suggest that MSCs help tumor cells evade the immune system by directly suppressing immune cells or by suppressing them indirectly via soluble factors [140]. They can also contribute to the development of new blood vessels that feed the tumor [141]. With regard to their anti-tumor effects, they can directly target and kill [9] cells and can be engineered to deliver anti-cancer therapies specifically to the tumor site [142]. Investigations into the roles of MSCs in cSCC remain in their nascent stages. A comprehensive understanding of the interactions between these cells and cSCC cells, as well as the broader tumor microenvironment, is essential before their potential as a viable cSCC treatment modality can be thoroughly evaluated.

### 4.3. Endothelial Cells

Endothelial cells, which constitute the lining of tumor blood vessels, play a pivotal role in delivering nutrients and oxygen to support neoplastic growth [10]. The expression of VEGF by these cells enhances vascular permeability [11]. This increased permeability leads to the extravasation of fluids and macromolecules, which facilitates tumor development and metastasis [12]. Within the TME, endothelial cells are essential mediators of angiogenesis. This process facilitates the development of a dedicated tumor vasculature, ensuring a continuous supply of oxygen and nutrients to support neoplastic proliferation [143]. Research has shown that the TME of cSCC often exhibits increased angiogenesis [144].

Research indicates that angiogenesis, driven by tumor cells secreting pro-angiogenic factors, represents an early and crucial hallmark of pre-malignant lesions. This increased vascularization, which is evident through elevated microvessel density, likely acts as a preparatory step facilitating further tumor progression. Understanding the dynamics of endothelial cell activity within pre-malignant stromal environments could provide valuable insights for developing diagnostic markers and early-stage therapeutic interventions against various cancers, including cSCC [145].

Another study examining eyelid lesions suggests a progressive increase in angiogenesis along the transition from pre-malignant lesions to invasive cancer. This is evidenced by heightened expression of VEGF and its receptors, which directly correlate with greater microvascular density within the TME. This trend intensifies in invasive cSCC, with larger tumors exhibiting markedly elevated levels of pro-angiogenic factors compared to pre-malignant lesions like actinic keratosis. These findings underscore the dynamic role of endothelial cells in shaping the TME, with angiogenesis becoming increasingly pronounced during the progression to malignancy [146].

### 4.4. Pericytes

Pericytes are contractile cells that wrap around the endothelial cells lining capillaries and venules, and they play a crucial role in blood vessel formation, stabilization, and blood flow regulation [147]. In the cSCC TME, pericytes display both pro-tumorigenic effects, by secreting VEGF and Platelet-Derived Growth Factor (PDGF) or by assisting the cancer cell migration and invasion, and anti-tumorigenic effects, by helping normalize the blood vessel and improving chemotherapy delivery [14]. 

Some studies show that pericytes may be loosely attached or entirely detached, leading to leaky and dysfunctional blood vessels within the tumor [15], which in turn may cause the tumor mass to grow and cancer cells to spread. 

More studies are needed to fully understand the complex interactions between pericytes and cSCC tumor cells.

### 4.5. Immune Cells

The cSCC TME is a dynamic battleground of the tumor and the body’s immune system. Many different types of immune cells play both pro-tumorigenic and anti-tumorigenic roles [148]. 

#### 4.5.1. Tumor-Infiltrating Lymphocytes (TILs)

**Figure 3:**
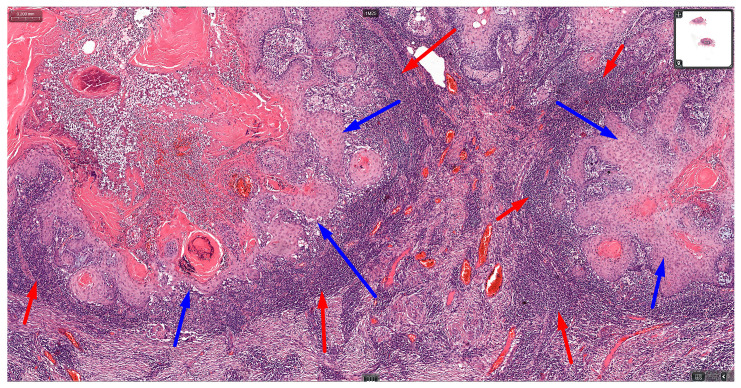
Photomicrograph of a squamous cell carcinoma (SCC) at 20× magnification, stained with hematoxylin and eosin (H&E). The blue arrows highlight islands of well-differentiated SCC with keratin pearls, a characteristic feature of this malignancy. The red arrows indicate a dense infiltrate of tumor-infiltrating lymphocytes (TILs) surrounding the tumor islands. The intervening stroma is densely collagenous and contains numerous blood vessels. Corner image: whole slide view of the tumor.

TILs have recently emerged as a significant prognostic factor in the management of various cancers, such as breast malignant neoplasm, melanoma, and squamous cell carcinoma of the head and neck region [149] (Figure 3). 

Firstly, they serve as an indicator of the host’s immune response to the malignancy. CD8+ cytotoxic T cells, a primary constituent of TILs, possess the capacity to identify and eliminate tumor cells [150] directly. Increased TIL density frequently signifies a more robust anti-tumor immune response [151]. Across diverse cancer types, research has broadly linked higher TIL levels with improved patient outcomes, including prolonged survival and decreased recurrence risk [152].

Secondly, TILs offer insights into the prevailing state of the TME. The composition of the TIL population is crucial. A predominance of CD8+ cytotoxic T cells indicates a potential for anti-tumor activity, whereas elevated levels of regulatory T cells (Tregs) suggest immunosuppression [153]. Furthermore, certain tumors possess an enhanced capacity to evade immune surveillance or directly suppress the immune response. In these cases, lower TIL densities may reflect the effectiveness of these tumor-driven evasion mechanisms [154].

The ratio between regulatory T cells (Tregs, CD4+) and cytotoxic T cells (CD8+) within the tumor microenvironment (TME) demonstrates substantial variability in cutaneous squamous cell carcinoma (cSCC). Several factors contribute to this heterogeneity, including tumor-specific characteristics and the host immune response.

Genetic mutations and the resulting tumor type heavily influence the immune profile [155]. Certain tumors exhibit an “immunogenic” nature, producing signals that recruit immune cells, while others actively establish an immunosuppressive TME [156]. The cancer stage is also crucial, as tumors acquire mechanisms to evade immune surveillance and promote immunosuppression throughout their progression [157].

A study investigating cSCC-development stages—which include normal skin, actinic keratosis, in situ cSCC, and invasive cSCC—found an increasing Treg populations in pre-invasive lesions and a decline in these populations in invasive cSCC [158]. Furthermore, Tregs were more frequent in indolent subtypes and pT2 tumors in comparison to pT1 and aggressive forms of tumors [158]. These dynamics hint at potential Treg exhaustion [159] or physical barriers within the tumor stroma, where high hyaluronic acid or collagen content might hinder Treg access to the microenvironment [108].

The burgeoning field of microbiome research reveals the skin microbiota’s potential to modulate immune responses. This community of microorganisms may directly interact with immune cells or release metabolites with immune-influencing properties, affecting anti-tumor activity and potentially immunotherapy outcomes [160].

Finally, TILs possess potential predictive value for the efficacy of immunotherapy. Checkpoint inhibitors function by reinvigorating a suppressed immune response [161]. Tumors exhibiting higher TIL densities may harbor pre-existing T cells that, upon release from inhibition, demonstrate an enhanced likelihood of responding to these therapeutic approaches [162].

In the context of cutaneous squamous cell carcinoma (cSCC), a study demonstrated that elevated numbers of CD8+ TILs (cytotoxic T cells, which play a critical role in direct tumor cell elimination) were observed in cSCCs lacking metastasis. These cSCCs were also more prevalent in sun-exposed anatomical locations, presented with smaller dimensions, and exhibited an overall lower frequency of genetic mutations [163]. Despite an elevated presence of CD8+ cytotoxic T cells, the immune system often fails to achieve complete clearance of cancer cells. This can be attributed to factors including T cell exhaustion, where chronic stimulation within the tumor microenvironment leads to a progressive loss of cytokine production and cytotoxic function [164]. Physical barriers within the tumor stroma, such as dense collagen or hyaluronic acid deposits, may also hinder T cell infiltration and access to tumor cells [108]. Additionally, tumor cells can evade immune surveillance by downregulating molecules crucial for immune recognition [159]. Finally, the nutrient-poor and acidic conditions within the tumor microenvironment (TME) can compromise T cell metabolism and anti-tumor activity [165].

#### 4.5.2. Tumor-Associated Macrophages (TAMs)

Tumor-associated macrophages (TAMs) represent an active area of investigation in cutaneous squamous cell carcinoma (cSCC) [166]. These versatile immune cells are frequently found within the cSCC tumor microenvironment (TME) [167]. Consistent with observations in other malignancies, TAMs in cSCC exhibit either a pro-inflammatory (M1) or pro-tumorigenic (M2) phenotype [168]. The presence of M2-polarized TAMs is often correlated with adverse clinical outcomes.

There is growing evidence suggesting that tumor-associated macrophages (TAMs) can be prognostic factors in various cancers, including cutaneous squamous cell carcinoma (cSCC) [168]. Studies suggest a higher density of TAMs within the tumor microenvironment is often associated with worse outcomes [169].

TAMs are incredibly plastic, and their role can be influenced by many factors within the tumor microenvironment [170]. Within TME, both tumor cells and surrounding immune cells release a diverse range of signaling molecules that influence the recruitment and polarization of TAMs. These signals can promote either a pro-tumor M2 phenotype (e.g., IL-4, IL-10, TGF-beta, and CCL2) [171] or can drive TAMs towards an anti-tumor M1 phenotype (e.g., IFN-gamma and TNF-alpha) [172]. Hypoxia, a frequent hallmark of solid tumors, can promote the development of an M2-like TAM phenotype [173]. Furthermore, tumors often exhibit metabolic reprogramming compared to healthy tissue [174]. Specific metabolites, such as lactate, can influence TAM function by driving them towards an immunosuppressive state [175]. The composition and biophysical properties of the extracellular matrix (ECM) and the structural scaffolding surrounding cells also modulate TAM behavior and motility [176]. Notably, the presence and composition of other immune cell populations within the TME can profoundly shape TAMs. For instance, regulatory T cells (Tregs) have been shown to promote M2 polarization [177]. It is essential to recognize that TAM polarization is not strictly confined to a binary M1/M2 paradigm [178]. In response to the complex cues within the TME, TAMs can display a spectrum of phenotypes [179]. Research continues to elucidate novel factors and mechanisms governing TAM behavior in the context of cancer.

TAMs play a crucial role in driving tumor angiogenesis, which is a key feature of cancer development [180,181]. In the hypoxic tumor microenvironment, TAMs release a diverse array of pro-angiogenic factors, including VEGF-A, SEMA family members, and S100 proteins, which stimulate new blood vessel formation [182,183]. Additionally, TAMs contribute to angiogenesis by secreting metalloproteinases (MMPs) that remodel the extracellular matrix, releasing endothelial cell mitogens and stimulating blood vessel growth indirectly through pro-inflammatory cytokine production [184]. Moreover, specialized TAM subpopulations like Tie2-expressing monocytes (TEMs) actively infiltrate tumors, promoting vascular development and potentially contributing to therapeutic resistance and tumor recurrence [183,184].

TAMs contribute to drug resistance through several mechanisms [170]. Their secretion of immunosuppressive cytokines (IL-10 and TGF-beta) hinders cytotoxic T cell function, compromising the effectiveness of immunotherapies [185]. Additionally, TAMs can form physical barriers around tumor cells, obstructing access for both therapeutic drugs and immune cells [186]. Moreover, TAMs promote drug resistance by inducing drug efflux pumps on cancer cells, facilitating the expulsion of chemotherapeutic agents [187]. TAMs can also alter drug metabolism within cancer cells, sometimes leading to treatment inactivation [188]. Furthermore, TAMs support the maintenance of cancer stem cells, which are inherently more drug-resistant than other cells, and which contribute to an overall immunosuppressive tumor microenvironment that diminishes the efficacy of various therapies [189].

TAMs actively promote tumor metastasis through a variety of mechanisms [190]. They induce epithelial–mesenchymal transition (EMT), which enables cancer cells to become migratory and invasive [191]. They can facilitate the entry of cancer cells into the circulation (intravasation) by degrading vascular barriers and promoting angiogenesis [192]. Within the bloodstream, TAMs protect circulating tumor cells (CTCs) from immune attack and aid their exit from blood vessels (extravasation) [192]. Furthermore, TAMs contribute to the formation of pre-metastatic niches at distant sites, creating a supportive environment for arriving cancer cells by secreting growth factors and cytokines and suppressing local immune responses [193,194]. Due to their pro-metastatic roles, TAMs are promising targets for anti-metastatic therapies using strategies focusing on TAM depletion, reprogramming, or blocking specific pathways involved in TAM-driven metastasis [195].

#### 4.5.3. Myeloid-Derived Suppressor Cells (MDSCs)

Myeloid-derived suppressor cells are immature myeloid cells that have potent immunosuppressive effects that hinder the ability of T cells to fight cancer [196]. Tumors often secrete a range of cytokines and chemokines that promote MDSC development and recruitment to the tumor microenvironment such as granulocyte-macrophage colony-stimulating factor (GM-CSF), which stimulates the generation of MDSCs from bone marrow precursors [197]; interleukin 6 (IL-6), which promotes MDSC expansion and survival [198]; and VEGF, which attracts MDSCs to the tumor site and promotes their survival [199]. 

Tumors can produce high levels of prostaglandins, particularly prostaglandin E2 (PGE2), which encourages the differentiation of myeloid cells into MDSCs [200]. The hypoxic environment common within tumors can stimulate the production of factors like HIF-1α (hypoxia-inducible factor 1-alpha), which promotes MDSC accumulation [201]. Tumors release small vesicles called exosomes that carry various molecules like proteins, RNA, and DNA. These exosomes can signal to bone marrow cells, stimulating the generation and expansion of MDSCs [202]. The altered metabolism of tumor cells (like the increased production of lactic acid) can create a microenvironment that favors MDSC development and function [203].

When specifically referring to cSCC, studies have shown that tumors that have features associated with a higher risk of metastasis also have a significantly increased presence of neutrophils and/or granulocytic myeloid-derived suppressor cells (G-MDSCs) [204]. This finding suggests that a specific immune cell profile within the tumor microenvironment might contribute to the aggressive behavior of high-risk cSCCs. The levels of certain immune cells, especially neutrophils and G-MDSCs, could potentially serve as biomarkers to help identify cSCCs with a greater likelihood of metastasis [204].

In another study, authors found that the neuropeptide methionine enkephalin (MENK) is highly expressed in human cSCC tissue [205]. MENK acts on opioid receptors in both MDSCs and tumor-associated macrophages (TAMs) to influence their functions within the tumor microenvironment. MENK appears to decrease the immunosuppressive abilities of MDSCs and shifts the polarization of TAMs from an immunosuppressive M2 phenotype to a more pro-inflammatory M1 phenotype [205].

#### 4.5.4. Natural Killer Cells (NK Cells)

Part of the innate immune system, NK cells provide rapid responses and can target tumor cells without prior sensitization [206]. These cells do not need prior exposure to a pathogen to recognize and destroy it (unlike T cells) [207]. NK cells play a critical role in the surveillance and elimination of tumor cells [208]. Research suggests that NK cells in the TME of cSCC are often reduced in number and have decreased functionality. This makes them less effective at identifying and killing tumor cells. cSCC cells can develop mechanisms to evade NK cell attack [209].

Studies show that NK cells can target and destroy cSCC cells [210]. Their presence can hinder the expansion and invasion of cSCC tumor cells [209]. NK cells directly trigger programmed cell death (apoptosis) in cSCC cells [210]. This research reinforces that a healthy immune system, specifically NK cells, plays a significant role in keeping cSCC growth in check.

Another study highlights the collaboration between NK cells and fibroblasts in controlling SCC invasion [211]. The CLEC2A molecule expressed by fibroblasts is essential for communication between these two cell types and enables NK cells to effectively regulate SCC invasion [211]. Patients with xeroderma pigmentosum, a genetic disease that weakens the skin’s defense against sun damage, might have impaired NK cell and fibroblast communication due to CLEC2A dysfunction. This could contribute to their increased risk of developing SCC [211].

#### 4.5.5. Dendritic Cells (DCs)

Dendritic cells (DCs) assume a vital role in anti-tumor immunity. They process and present tumor antigens to T cells, facilitating the activation of a cytotoxic immune response [212,213]. Several DC subtypes reside in human skin: Langerhans Cells (LCs), in the epidermis; Dermal Dendritic Cells (DDCs), in the dermis; and Plasmacytoid Dendritic Cells (pDCs) [214,215,216].

A study has demonstrated impaired DC function within cutaneous squamous cell carcinoma (cSCC). This impairment manifests as reduced quantities of LCs and DDCs within cSCC lesions, impacting the anti-tumor immune response [216]. Moreover, tumor-associated myeloid DCs exhibit diminished T cell stimulatory capacity. Evidence suggests that this dysfunction may partially arise from a suppressive tumor microenvironment characterized by elevated cytokines (like IL-10 and TGF-β) that inhibit DC maturation [216,217]. Additionally, increased quantities of regulatory T cells (Tregs) contribute to DC dysfunction and suppress CD8+ cytotoxic T cell activity against the tumor. Furthermore, TIP-DCs (CD11c+ mDCs) within the tumor microenvironment (TME) may exert direct immunosuppressive effects. Their secretion of TNF and iNOS catalyzes nitric oxide (NO) production, which subsequently inhibits activated T cell proliferation [218].

Separate findings further support this notion of disrupted DC function in cSCC, specifically demonstrating hindered myeloid DC ability to activate T cells [218].

LCs present a paradoxical role in the cSCC tumor stroma: enhanced T cell-activation ability is observed in vitro, yet ineffective tumor growth prevention is observed in vivo. This discrepancy might be attributed to factors such as reduced LC numbers or impaired migration and T cell-activation capacity within lymph nodes [216].

In contrast, plasmacytoid DCs within cSCC lesions hold the potential for anti-tumor activity due to their IFN-α production capability, though ongoing research is needed to fully elucidate their role [216].

A comprehensive understanding of the complex cSCC tumor environment remains crucial for developing strategies to restore dendritic cells’ anti-cancer activity.

### 4.6. Adipocytes

**Figure 4:**
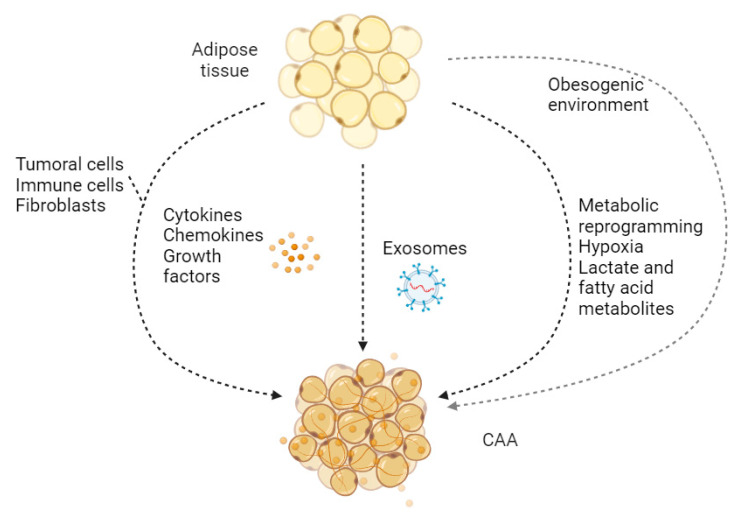
Transformation of adipocytes within the tumor microenvironment.

Adipocytes are fat cells that can store energy and produce various factors promoting tumor growth and metastasis [219] (Figure 4). They have important endocrine functions, secreting hormones (adipokines) such as leptin and adiponectin [220]. In the TME, they undergo changes and become what are known as cancer-associated adipocytes (CAAs) [221], which differ from normal adipocytes in several ways. For instance, CAAs have a different phenotype from normal adipocytes, the first being smaller than the latter due to lipolysis [222]. Their metabolism is also altered, with CAAs exhibiting increased fatty acid release, that provides cancer cells with fuel for growth and invasion [223], and they are able to secrete different profiles of adipokines and inflammatory molecules that influence the TME [224].

In the TME, these cells serve as fuel providers for the cancer cells. The breakdown of fat and release of fatty acids by CAAs directly provides tumor cells with the energy to grow and spread [225]. They also contribute to a chronic inflammatory state within the TME, which can aid cancer progression and suppress immune responses [226]. CAAs influence the recruitment and function of immune cells, potentially leading to an immunosuppressive environment that allows the tumor to evade immune attack [227]. Moreover, CAAs may facilitate cancer cell invasion and distant spread [223].

Obesity is strongly associated with an increased risk of several cancers, and adipocytes play a significant role in this connection [228]. Increased levels of adipose tissue in obesity leads to a greater abundance of CAAs in the TME, exacerbating their adverse effects [222].

While the role of cancer-associated adipocytes has been elucidated in several cancer types, their specific contributions within the tumor microenvironment of cutaneous squamous cell carcinoma remain largely unexplored. The proximity of subcutaneous adipose tissue to cSCC lesions suggests a potential interaction, warranting further investigation. Studies focused on characterizing adipocytes near cSCC tumors and determining whether they exhibit phenotypic changes consistent with CAAs would be essential in delineating the functional influence of these cells in cSCC development and progression.

The complex interplay of tumor-derived factors shapes the transformation of normal adipocytes into cancer-associated adipocytes (CAAs) within the tumor microenvironment (TME). Cancer cells release cytokines, chemokines, growth factors, and exosomes that influence adipocyte gene expression, metabolism, and secretory profiles. Chronic inflammation further amplifies these signals, allowing them to be fueled by immune cells within the TME. The unique metabolic conditions and obesogenic environment can enhance adipocyte susceptibility to this reprogramming. The resulting CAAs exhibit altered characteristics such as decreased size, increased lipolysis, and the release of factors that promote tumor growth, angiogenesis, and immune suppression within the TME.

## 5. Targeting the Tumor Microenvironment: Advancing Cancer Therapy beyond Traditional Approaches

The tumor stroma represents a complex and dynamic microenvironment [229]. Its components are essential for cancer cell survival, growth, invasion, and metastasis [3]. Historically, cancer research and therapy have focused primarily on the malignant cells themselves, often neglecting the critical supportive role played by the surrounding stroma [229]. 

We understand now that the TME is far from a passive bystander [230] and that it plays a key role in helping cancer cells to evade the immune system. Thus, immunotherapy and boosting of the body’s anti-tumor response are important in cancer therapy [231]. Adoptive cell transfer, which involves removing, modifying, and re-infusing a patient’s immune cells to make them better at targeting cancer, has been used in other skin cancers, such as melanoma [232]. Immune checkpoint inhibitors are a class of drugs that target regulatory pathways critical for maintaining immune self-tolerance [233]. By inhibiting these immune checkpoint pathways, these therapies can restore the immune system’s capacity to identify and eradicate malignant cells [234]. Thus, drugs such as Cemiplimab (anti-PD-1) have shown promising results in the treatment of advanced stages of cSCC, especially for metastatic or locally advanced tumors [235,236]. It is important to consider, however, that immune checkpoint blockade (ICB) therapy can precipitate immune-related adverse events (irAEs) stemming from increased immune activity [237]. These irAEs may require close monitoring and management [238].

Targeting tumor angiogenesis represents another promising therapeutic strategy in cancer treatment [239]. Anti-angiogenic therapies, which are designed to disrupt the formation of new blood vessels, can limit a tumor’s access to nutrients and oxygen, potentially inhibiting growth and metastasis [240].

As we have seen before, CAFs constitute a major cellular component of the tumor stroma, playing a critical role in the establishment of a pro-tumorigenic microenvironment [241]. Promising therapeutic avenues should focus on targeting CAFs within the tumor stroma. These might include approaches aimed at depleting CAFs via selective targeting, reprogramming CAFs to diminish their tumor-supportive phenotype, disrupting CAF-mediated signaling pathways essential for tumor promotion, or targeting CAF-secreted factors that contribute to pro-tumorigenic remodeling of the extracellular matrix (ECM).

The ECM should also be a focus for therapy because it actively signals to cells, influencing tumor growth, metastasis, and response to treatment [242]. Tumor-specific ECM gene signatures can help to predict outcomes and sensitivity to treatment [243]. Breaking down ECM rigidity can enhance drug and immune cell infiltration. Enzymes like MMPs, LOX, and LOXLs modify ECM structure and are potential therapeutic targets [242]. Vitamin D has been shown to inhibit the generation of fibrosis-related ECM components [244]. While its precise mechanisms in cancer are still being researched, Vitamin D analogs show promise in normalizing cancer-associated ECM remodeling [242].

Targeting the interaction between ECM proteins and cell surface receptors represents a promising avenue for disrupting tumor-promoting signaling pathways. Antibodies designed to block specific ECM receptors can inhibit pro-tumorigenic signals, demonstrating potential for therapeutic intervention [242]. RGD motifs (Arginine-Glycine-Aspartic), designed for integrin recognition, can be harnessed for integrin inhibition, targeted drug delivery, or tumor imaging applications [242,245]. Additionally, receptors such as discoidin domain-containing receptors (DDRs), CD44, and syndecans offer further therapeutic targets for domain-specific blockade [242,246,247]. Strategic disruption of secreted ECM proteins, like those containing the fibrinogen-like globe (FBG) domain, is another potential approach to hindering tumor growth [248]. Paradoxically, the very ECM components fueling tumor progression can be exploited as targets for drug delivery. This includes systems using fibronectin components [249] or nanoparticles designed for ECM penetration [250].

Chimeric antigen receptor (CAR) T cell therapy offers a promising treatment modality demonstrating notable efficacy against hematologic malignancies [251]. Compared to monoclonal antibodies, CAR-T cells exhibit enhanced tumor infiltration capabilities [242]. However, their limited success in solid tumors highlights the need to overcome the extracellular matrix (ECM) barriers within the tumor microenvironment [252]. Strategies to enhance CAR-T penetration include engineering cells to overexpress heparinase for ECM degradation [253] or combining them with oncolytic viruses that facilitate ECM remodeling [242]. Modifying the epigenetic landscape of CAR-T cells may promote a favorable memory phenotype and increase their overall therapeutic efficacy [254]. Additionally, targeting stromal cells, particularly cancer-associated fibroblasts, which are primary producers of ECM components, represents another avenue for ECM disruption. Fibroblast activation protein (FAP), which is overexpressed on CAFs, serves as a potential target for CAR-T therapies designed to degrade the ECM [255]. Research exploring CAR-T cells for ECM remodeling is still in its early stages, and data regarding cutaneous squamous cell carcinoma (cSCC) is particularly scarce. This emphasizes the need for focused research efforts to translate the promise of CAR-T therapies into effective treatments for cSCC.

Inter-patient variation in the TME is likely to be observed in cutaneous squamous cell carcinoma, even among individuals presenting with the same stage of disease. This heterogeneity arises from a complex interplay of factors, including genetic polymorphisms affecting innate and adaptive immune responses [256], environmental exposures (such as UV radiation) with immunomodulatory effects [257], intratumoral heterogeneity that influences signaling pathways [258], the specific anatomical location of the tumor, and the patient’s overall health status, which can further shape systemic immune competence [259].

This variability of the TME in cSCC strongly suggests the potential for personalized therapeutic approaches. As TME composition influences treatment responses, a standard approach is unlikely to be universally effective [260]. By characterizing the specific TME of a patient’s tumor, including its immune cell profile and signaling pathways, clinicians might tailor therapies accordingly [261]. Additionally, combining angiogenesis inhibitors with TME-altering strategies holds promise [262] [G]. While the complexity and dynamic nature of the TME poses challenges, well-designed clinical trials evaluating the efficacy of TME-based personalized therapies are essential for demonstrating their superiority over standard approaches and ultimately revolutionizing cSCC patient care.

## 6. Conclusions

The tumor microenvironment, like a vast and intricate micro-universe, teems with dynamic interactions between its diverse components and the malignant cells themselves. While much remains to be discovered about this complex system, its potential as a target for novel anti-cancer therapies offers a beacon of hope. Further research is crucial in order to illuminate these relationships and harness the full therapeutic potential hidden within this complex cellular landscape.

From our perspective, the promise of personalized treatments based on TME profiling is tempered by concerns about feasibility and accessibility. While TME analysis techniques are rapidly improving, they currently require specialized skills and can be expensive, which could potentially limit their widespread use. Furthermore, developing and manufacturing therapies tailored to individual TME profiles is likely to be more costly than standard treatments. This raises significant questions about affordability and equitable access for patients. The dynamic nature of the TME also necessitates regular monitoring, adding complexity and further straining resources. Despite these challenges, TME-tailored therapies hold immense potential. Addressing the logistical and economic hurdles will be critical in ensuring that this promising approach becomes widely feasible and accessible, allowing all patients to benefit.
